# Endovascular Treatment of Acute Stroke Due to Intracranial Atherosclerotic Stenosis–Related Large Vessel Occlusion

**DOI:** 10.3389/fneur.2019.00308

**Published:** 2019-04-02

**Authors:** Hyungjong Park, Jang-Hyun Baek, Byung Moon Kim

**Affiliations:** ^1^Interventional Neuroradiology, Department of Radiology, Severance Stroke Center, Severance Hospital, Yonsei University College of Medicine, Seoul, South Korea; ^2^Department of Neurology, Keimyung University School of Medicine, Daegu, South Korea; ^3^Department of Neurology, Kangbuk Samsung Hospital, Sungkyunkwan University School of Medicine, Seoul, South Korea

**Keywords:** acute stroke, large vessel occlusion, intracranial atherosclerosis, endovascular treatment, stenosis and cerebrovascular occlusion

## Abstract

Endovascular treatment (EVT) has become a standard treatment for acute ischemic stroke due to large vessel occlusion (LVO) in the anterior circulation. However, whether EVT tools used for intracranial atherosclerotic stenosis (ICAS)-related LVO are as safe and effective as for use in embolic LVO remains unclear. There have been only a few studies about EVT for ICAS-related LVO, and these studies revealed that mechanical thrombectomy with a stent retriever or contact aspiration was less effective and more time consuming in ICAS-related LVO than in embolic LVO. Because fast and successful recanalization (defined as modified Thrombolysis in Cerebral Ischemia grade, 2b or 3) is the most critical factor influencing favorable outcomes, it is important to determine the appropriate EVT strategy for fast recanalization of ICAS-related LVO. In this report, we review the results of mechanical thrombectomy using stent retriever or contact aspiration and rescue treatments after failure of mechanical thrombectomy for ICAS-related LVO. Finally, we propose the EVT strategy appropriate for ICAS-related LVO based on a literature review and our experience.

## Introduction

Endovascular treatment (EVT) for acute stroke due to emergent large vessel occlusion (LVO) has been successful, and EVT has become a standard treatment for LVO (1). The rate of recanalization, defined as modified Thrombolysis In Cerebral Ischemia (mTICI) grade 2b or 3, has been improving since the first 5 successful EVT trials, which demonstrated that higher recanalization rates are associated with better clinical outcomes (2–5). For mechanical thrombectomy, the two mainstay modalities are stent retriever (SR) and contact aspiration (CA) thrombectomies. Both methods were primarily invented for removal of the embolic clots occluding the large vessel. The pathomechanism of intracranial atherosclerosis (ICAS)-related LVO is likely due to *in-situ* thromboocclusion rather than embolic occlusion (6–8). The efficacy of both methods for recanalization of *in-situ* thromboocclusion in ICAS-related LVO has not been well elucidated. ICAS is one of the main causes of acute stroke in Asian, Hispanic, and African populations (9–11) and furthermore recent studies have documented that ICAS-related LVO is responsible for approximately 12–30% of all causes of LVO in East Asia (7, 8, 12–16). Therefore, it is worthwhile to investigate the efficacy of SR or CA thrombectomy and the most appropriate EVT strategy for ICAS-related LVO. This review aims to investigate on what are the problems in EVT and to find out the appropriate treatment strategy for ICAS-related LVO.

## Outcomes of Endovascular Treatment for ICAS-Related LVO

Until recently, there have been only a few retrospective studies in which the clinical outcomes of EVT for ICAS-related LVO have been evaluated, and the results were inconsistent. In a study, patients with ICAS-related LVO had more favorable outcomes than patients with embolic LVO (15). In contrast, other researchers showed less favorable outcomes or no significant difference between the two groups (8, 14, 16–18). Notably, the rate of favorable outcomes was proportionate to the recanalization success rate. Successful recanalization and favorable outcome rates were observed more frequently in patients with ICAS-related LVO in a few reports, but the opposite was observed in a different study. In another study with similar recanalization rates, clinical outcomes were similar between the two groups (Table 1).

**Table 1:** Comparison of outcomes between intracranial atherosclerotic stenosis-related and embolic large vessel occlusion.

**References**	**Group**	**Recanalization**	**mRS, 0–2**	**Symptomatic ICH**	**Mortality**
Kang et al. (8)	ICAS-related	80.0%	65.0%	2.5%	NA
	Embolic	83.7%	67.5%	5.4%	NA
Lee et al. (16)	ICAS-related	62.5%	41.7%	NA	8.3%
	Embolic	63.6%	31.3%	NA	24.6%
Jia et al. (18)	ICAS-related	95.7%	63.8%	4.3%	12.8%
	Embolic	96.8%	51.6%	4.3%	12.9%
Al Kasab et al. (17)	ICAS-related	^*^64.7%	^*^42.4%	11.1%	39.4%
	Embolic	^*^95.3%	^*^55.8%	9.8%	20.7%
Yoon et al. (15)	ICAS-related	^*^95.0%	^*^62.5%	7.5%	15.0%
	Embolic	^*^81.8%	^*^38.6%	3.0%	9.1%
Baek et al. (14)	ICAS-related	80.4%	46.4%	5.4%	19.6%
	Embolic	88.5%	46.9%	5.0%	15.3%
Lee et al. (19)	ICAS-related	76.8%	45.5%	7.1	NA
	Embolic	79.6%	54.5%	10.7	NA

ICAS, intracranial atherosclerotic stenosis; mRS, modified Rankin Scale score; ICH, intracranial hemorrhage;

* *, statistically significant*.

In a recent study, ICAS-related LVO was a worse prognostic factor in multivariate analysis although recanalization rates were similar between embolic and ICAS-related LVO groups. A significant interaction of underlying etiology (ICAS verse embolic) on patient outcome was observed with procedure (puncture-o-recanalization) time. Therefore, they suggested that “the relatively poor outcome in the ICAS-related LVO is mainly attributable to longer procedure time, reflecting the procedure complexity and the higher rate of reocclusion” (19).

From the results of the previous studies, we can infer that recanalization status and procedural time (puncture-to-recanalization time) are more relevant factors affecting patient outcomes than occlusion etiology itself (ICAS vs. embolic). In other words, if successful recanalization in the ICAS-related LVO was achieved as fast and with high rate as in the embolic LVO, functional outcome of ICAS-related LVO would be comparable to that of embolic LVO.

## Endovascular Treatment for ICAS-Related LVO

### Stent Retriever Thrombectomy for ICAS-Related Large Vessel Occlusion

SR thrombectomy is recommended as the first-line EVT modality for acute stroke due to anterior circulation LVO (1). SR thrombectomy for obtaining initial recanalization appeared to be as effective in ICAS-related LVO as in embolic LVO (6, 7, 12–16, 19). However, reocclusion during EVT is very frequent after an initial recanalization with SR thrombectomy in ICAS-related LVO, with reported ranges from 57.1 to 77.3% (6, 7, 12–16). For SR thrombectomy, a microwire should be passed through the occlusion site followed by a microcatheter with an inner diameter ≥0.021-inch. Although physicians may be concerned that passage of a microwire followed by a microcatheter is potentially dangerous, the initial SR attempt was successful in most ICAS-related LVO cases, and has not been reported to be difficult (6, 7, 13–15). However, once reocclusion occurs, repeated SR thrombectomy attempt seemed to be ineffective and prone to procedural complications because it may cause vessel injury, resulting in repeat reocclusion, spasm, and dissection (6, 7, 13–16, 20, 22, 23).

**Figure 1:**
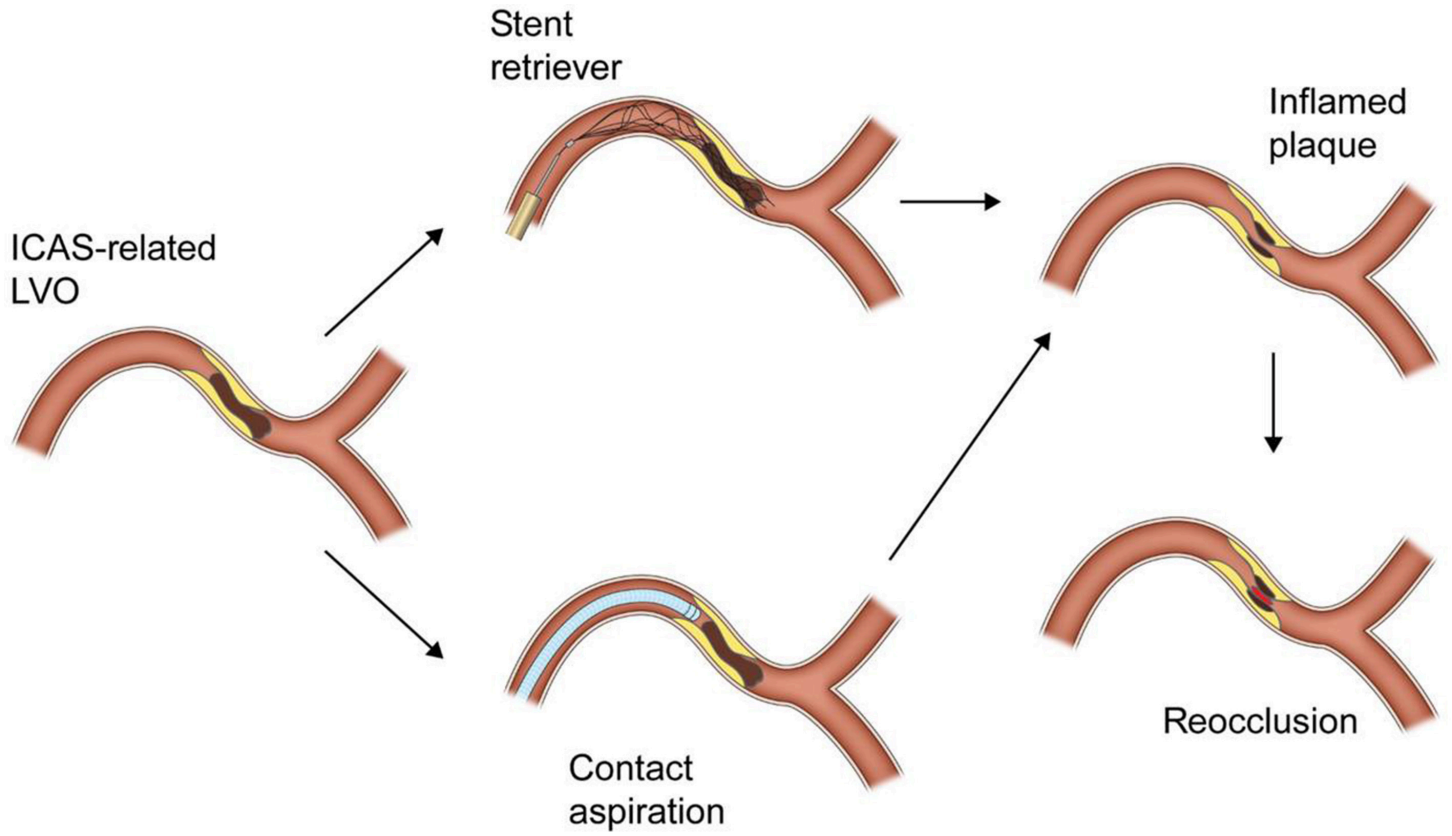
A schematic drawing of reocclusion mechanism after stent retriever or contact aspiration thrombectomy for intracranial atherosclerotic stenosis-related large vessel occlusion.

Although it has not yet been well known why reocclusion is so frequent in ICAS-related LVO, it can be explained by its pathomechanism and thrombectomy procedure itself. The pathomechanism of ICAS-related LVO, similar to that of coronary artery disease, is likely *in-situ* thromboocclusion due to unstable plaque rupture (6–8). An acute *in-situ* clot formed on an ICAS plaque is platelet-rich one (22). Repeat passages of a microwire, a microcatheter and SR may further damage the inflamed plaque and thus provoke more platelet activation and even arterial dissection (Figure 1) (6, 7, 21). Therefore, repeat attempts of SR thrombectomy should be avoided so far as possible once reocclusion has occurred in ICAS-related LVO (6, 7, 13, 14).

### Contact Aspiration Thrombectomy for ICAS-Related Large Vessel Occlusion

Although first-line CA thrombectomy is as effective as SR thrombectomy in embolic LVO, it seemed less effective for recanalization of ICAS-related LVO. In a study that compared the efficacy of SR vs. CA thrombectomy in 146 ICAS-related LVO, the rate of switching to alternative thrombectomy technique (SR to CA thrombectomy or vice versa) after the frontline modality failed was significantly higher in the CA group (40%) than in the SR group (4.7%, *p* < *0.001*) (21). Theoretically, a successful CA thrombectomy requires firm engagement of a large-bore catheter distal tip with the occluding clot. In ICAS-related LVO, the parent artery is likely to be tortuous in form and tapered to the occlusion site.

Therefore, the distal tip of a large-bore catheter is likely to less firmly engage the occluding clot in ICAS-related LVO than the embolic clot in a normal parent artery (Figure 1). Furthermore, the reocclusion rate of CA thrombectomy was similar to that of SR thrombectomy (8). In CA thrombectomy, a microcatheter and a microwire were navigated through the occlusion site and then a large-bore catheter is advanced over the wire and microcatheter to the occluding clot. Thus, the preceding passage of a microwire and a microcatheter may also irritate the inflamed plaque and eventually provoke more platelet activation in ICAS-related LVO.

### Rescue Treatments

**Figure 2:**
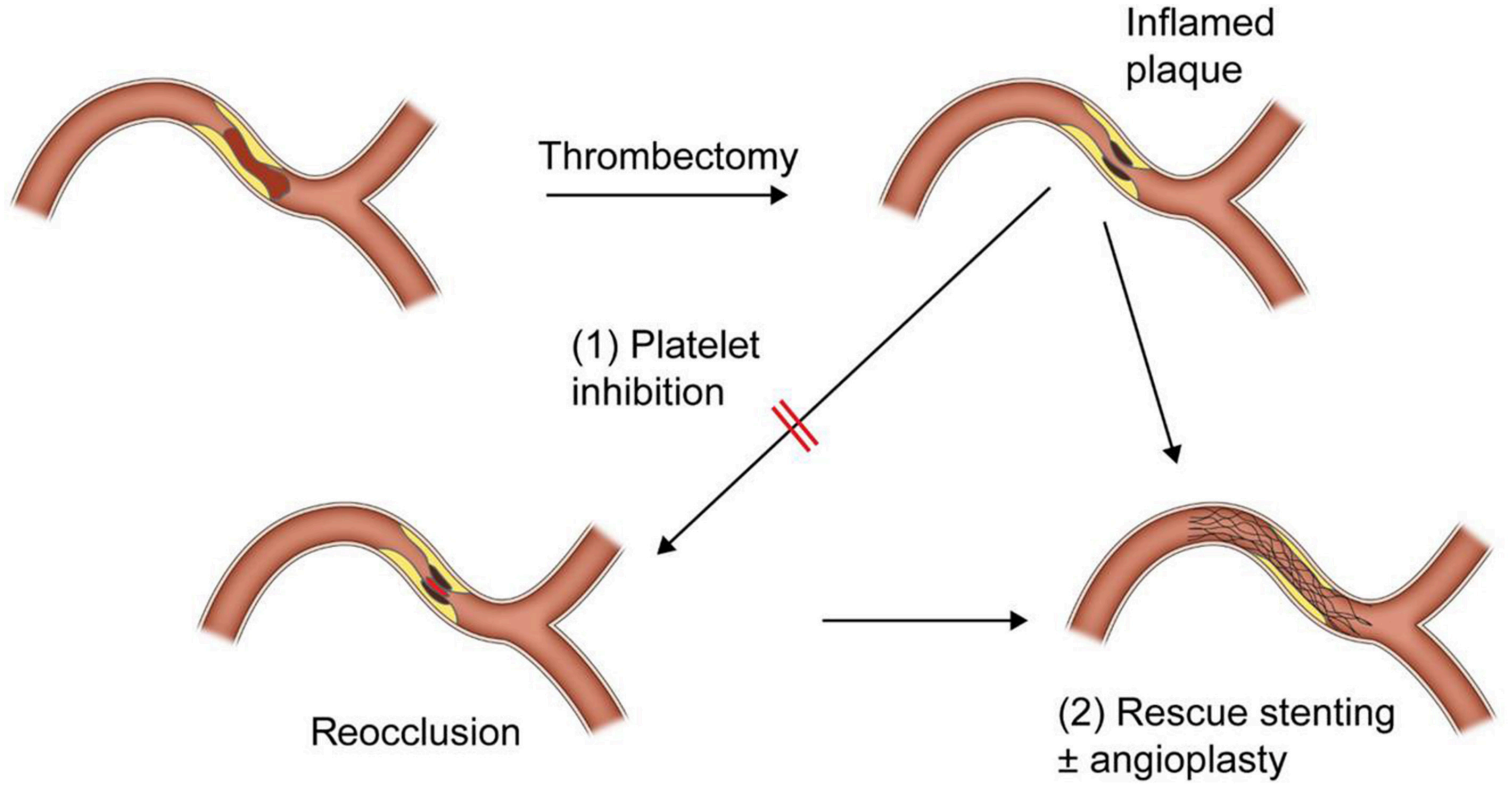
A schematic drawing of rescue treatment for reocclusion after initial recanalization of intracranial atherosclerotic stenosis-related large vessel occlusion.

As in the embolic LVO, fast and successful recanalization is the most important factor for achieving good outcomes in ICAS-related LVO. Therefore, if the primary thrombectomy failed to achieve recanalization, rescue treatments should be performed as soon as possible. Rescue treatments after failure of frontline thrombectomy for LVO include switching to another tool (SR to CA thrombectomy or vice versa), simultaneous use of both SR and CA thrombectomies, intraarterial thrombolytic infusion, intraarterial, or intravenous glycoprotein IIb/IIIa inhibitor (GPI) infusion and stenting with or without balloon angioplasty (6–8, 13–15, 18, 23–26). Because reocclusion in ICAS-related LVO is very frequent and most likely due to platelet activation, a severe degree of residual stenosis, or combined contribution of both, rescue treatment should be focused on platelet inhibition and alleviating the degree of residual stenosis. GPI has been suggested for platelet inhibition in the several previous reports. Intraarterial or intravenous infusion of low dose of GPI (Tirofiban, 0.5–1.5 mg or Reopro, 3–10 mg) was effective for resolution or prevention of re-occlusion of ICAS-related LVO (7, 8, 14) (Figure 2). GPI could make the endothelium more stable, which could reverse the *in-situ* thrombotic reocclusion tendency (6, 7, 14, 22). In our center, intra-procedural administration method of GPI is typically as follows; angiogram is obtained every 10 min at least 2 times after administration of 0.3–0.5 mg of Tirofiban (or Reopro, 3–5 mg). If reocclusion (tendency) is not resolved, additional 0.3–0.5 mg of Tirofiban (or Reopro, 3–5 mg) is administered up to 1.5 mg (or Reopro, 10 mg) until reocclusion (tendency) is resolved (or improved antegrade flow velocity) on angiogram obtained every 10 min. The dose of GPI did not depend on whether or not to use intravenous tPA administration.

**Figure 3:**
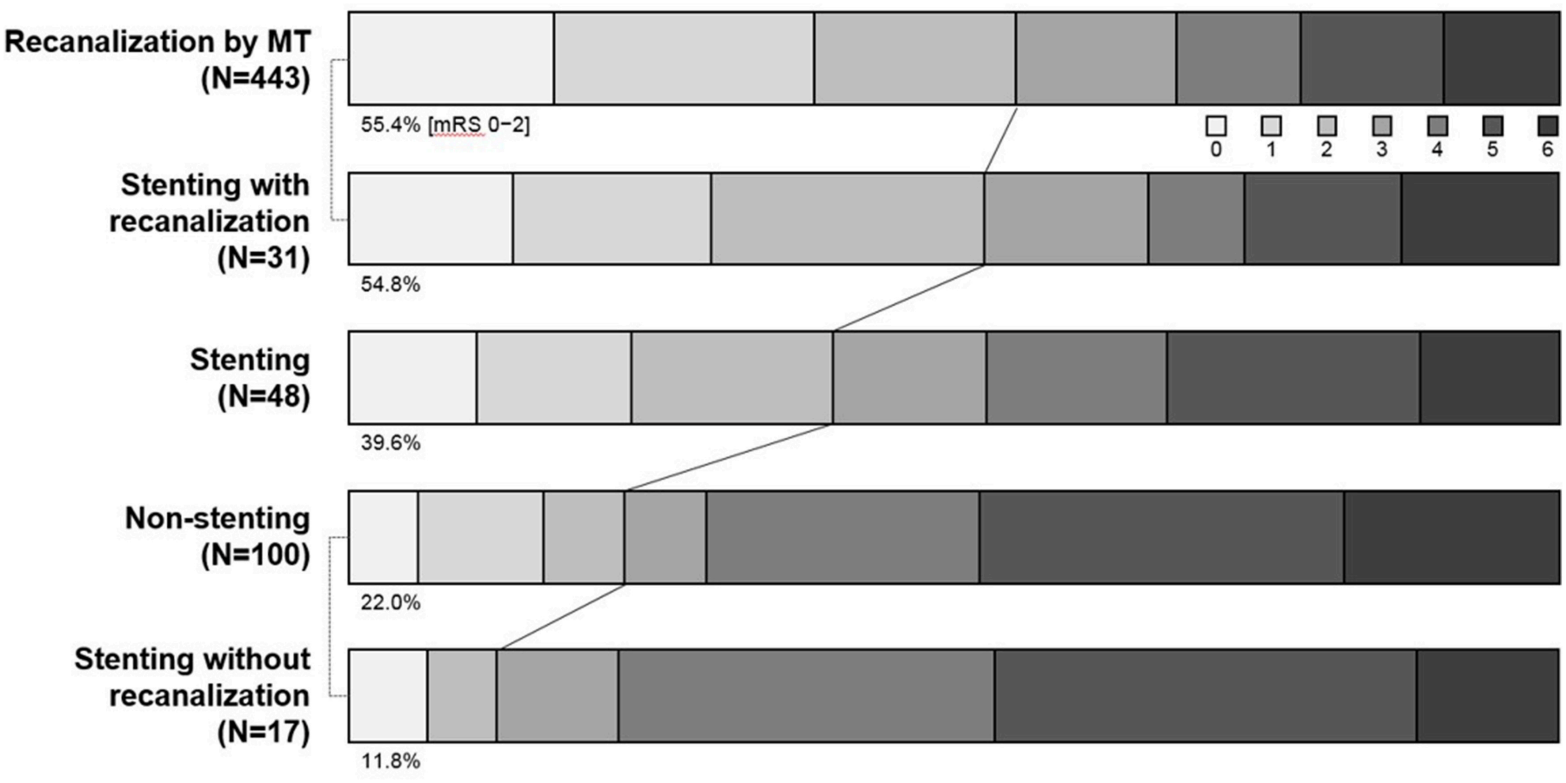
Comparison of modified Rankin scale score among mechanical thrombectomy success, rescue stenting, and non-stenting groups. MT, mechanical thrombectomy.

Rescue stenting and/or angioplasty is an another (or additional) option for resolving reocclusion. As well known, a severe degree of focal stenosis is an important factor that provokes clot formation. Therefore, by alleviating the underlying severe degree of stenosis, rescue stenting and/or balloon angioplasty is likely to play a role in preventing reocclusion or in recanalization for such cases that are never opened with mechanical thrombectomy (24, 25) (Figure 3). In literature, it seemed to depend on operators' preference whether to do stent alone, stent with pre- or post-stent balloon angioplasty, or balloon angioplasty alone (6, 17–19, 24, 25). It also depended on operators' preference to use what kind of stent. However, self-expanding stent seemed to be favored rather than balloon-expandable stent. In addition, of the self-expanding stent, Solitaire-FR stent rather than Wingspan stent seemed to be favored for rescue stenting. It is likely because Wingspan stent requires additional preparation time and more technical demands for delivery, whereas it seems simple to detach a Solitaire-FR that was already in using for thrombectomy (6, 24, 25).

Recent two studies compared patients who received rescue stenting with those who was left without further treatment after mechanical thrombectomy failure. Rescue stenting group showed better functional outcome than non-stenting group (24, 25). Patients with recanalization success showed a similar distribution of mRS at 3 months, regardless of recanalization methods (mechanical thrombectomy or rescue stenting) (Figure 3). In those studies, the majority of patients with rescue stenting had ICAS-related LVO. Therefore, rescue stenting may be a rescue method in ICAS-related LVO. Another recent study also have demonstrated that both GPI and rescue stenting are similarly effective and safe in ICAS-related LVO (23).

Postprocedural delayed reocclusion also worsen patients' functional outcome (25, 26). Timing and maintenance of postprocedural antiplatelet medication is important to prevent postprocedural reocclusion. Although there has not been controlled study, intravenous infusion of GPI with maintenance dose for a 6–12 h after completion of EVT and then change to oral antiplatelet medication has been proposed (14). The degree of residual stenosis may be an another factor for post-procedural reocclusion. It has not yet been studied whether rescue stenting and/or angioplasty is needed to prevent postprocedural delayed reocclusion. However, if the degree of stenosis is very severe, alleviating the stenosis by rescue stenting and/or angioplasty may be helpful in both instant and delayed reocclusion. It should be addressed if rescue stenting and/or angioplasty is helpful for preventing postprocedural delayed reocclusion.

It has yet remained unclear whether stenting combined with anti-thrombotic drug is safe in acute stroke setting. In recent reports, however, intracranial or cervical carotid artery stenting with use of antithrombotic medication in acute stroke setting did not increase the development of symptomatic intracranial hemorrhage, while significantly improved functional outcome (24, 25, 27). Because of retrospective nature of these studies, the type and mode of antithrombotic medication combined with stenting varied depending on centers; (1) intra-arterial or intravenous loading of GPI followed by maintenance of 6–12 h, then changed to oral dual antiplatelets, (2) loading dose of oral dual antiplatelets just before or after rescue stenting (aspirin 85–500 mg and clopidogrel 300 mg), (3) oral mono antiplatelet (clopidogrel, 75–300 mg), and (4) no antiplatelet medication until follow-up CT or MR on the next day (24, 27). When GPI and rescue stenting which requires antithrombotic medication is applied in acute stroke setting, the most feared concern is possibly increased risk of intracranial hemorrhage. In recent multicenter studies, however, intracranial or cervical carotid stenting combined with use of antithromboics drugs in acute stroke setting did not increase the development of symptomatic intracranial hemorrhage whereas significantly improved functional outcome (24, 25, 27). In our experience, it seems very helpful in decision making to obtain flat-panel CT before GPI infusion and/or rescue stenting. Although a specific criterion of unfavorable candidate for GPI and/or rescue stenting has not yet been recommended, we have not done further treatment for cases that have large area of contrast material enhancement on flat-panel CT. In this strategy, the rate of symptomatic intracranial hemorrhage was not higher in patients with GPI and/or rescue stenting than in patients without (7, 14, 24, 25).

## Endovascular Strategy Appropriate for ICAS-Related LVO

Recanalization status and procedural time are more relevant factors affecting patient outcomes than occlusion etiology. For faster and more successful recanalization, identifying underlying ICAS as the cause of LVO and setting an optimal strategy for ICAS-related LVO are key factors leading to better clinical outcomes in ICAS-related LVO patients. Before starting EVT, the following markers may suggest ICAS-related LVO from embolic LVO: (1) the absence of atrial fibrillation on echocardiogram and (2) absence of hyperdense artery sign on CT or susceptibility (blooming) artifact sign on MR gradient echo image, and (3) truncal-type occlusion on CTA. During the EVT, (1) a truncal-type occlusion and (2) a remnant (fixed focal) stenosis after initial recanalization at the occlusion site are suggested as useful surrogate markers of ICAS-related LVO (Table 2). It is critical to determine when to introduce what kind of rescue treatment after failure of mechanical thrombectomy in ICAS-related LVO. Procedural time in the ICAS-related LVO was consistently longer than in the embolic group across the all previous studies (7, 8, 12–16, 20, 21), and this can be explained by repeat SR or CA thrombectomy attempts before applying the appropriate rescue treatment for ICAS-related LVO (7, 12, 14, 19). If the appropriate EVT strategy for ICAS-related LVO can be set early (before starting EVT, if possible), operators can shorten the puncture–to-recanalization (procedural) time as well as increase recanalization rate, thus providing a better clinical outcome (6, 7, 14, 19). Rescue stenting and/or balloon angioplasty, intra-arterial or intravenous GPI infusion, or combination of those have been reported as appropriate treatments for ICAS thrombo-occlusion (7, 8, 14, 15, 18, 23, 23–25). The stepwise approach in using such modalities in addition to mechanical thrombectomy is likely helpful in faster and more successful recanalization of ICAS-related LVO.

**Table 2:** Surrogate markers suggested of intracranial atherosclerosis-related large vessel occlusion.

	**Surrogate markers**	**ICAS-related**	**Embolic**
Before starting EVT	Atrial fibrillation, 2017 (13)	+^*^	+++
	Susceptibility artifact on MR gradient echo image, 2015 (28)	+	+++
	Hyperdense artery sign on NECT, 2017 (29)	+	++
	Truncal-type occlusion on CT angiography, 2017 (13), 2016 (14)	+++	+
During the EVT	Truncal-type occlusion, 2016 (7), 2017 (13), 2016 (14)	++++	+
	Residual stenosis, 2014 (8), 2015 (16), 2018 (23)	++++	+

*EVT, endovascular treatment; ICAS, intracranial atherosclerotic stenosis; MR, magnetic resonance; NECT, non-enhanced computed tomography; CT, computed tomography*.

* *The number of + sign indicates to have more probability to have ICAS-related or embolic surrogate markers*.

**Figure 4:**
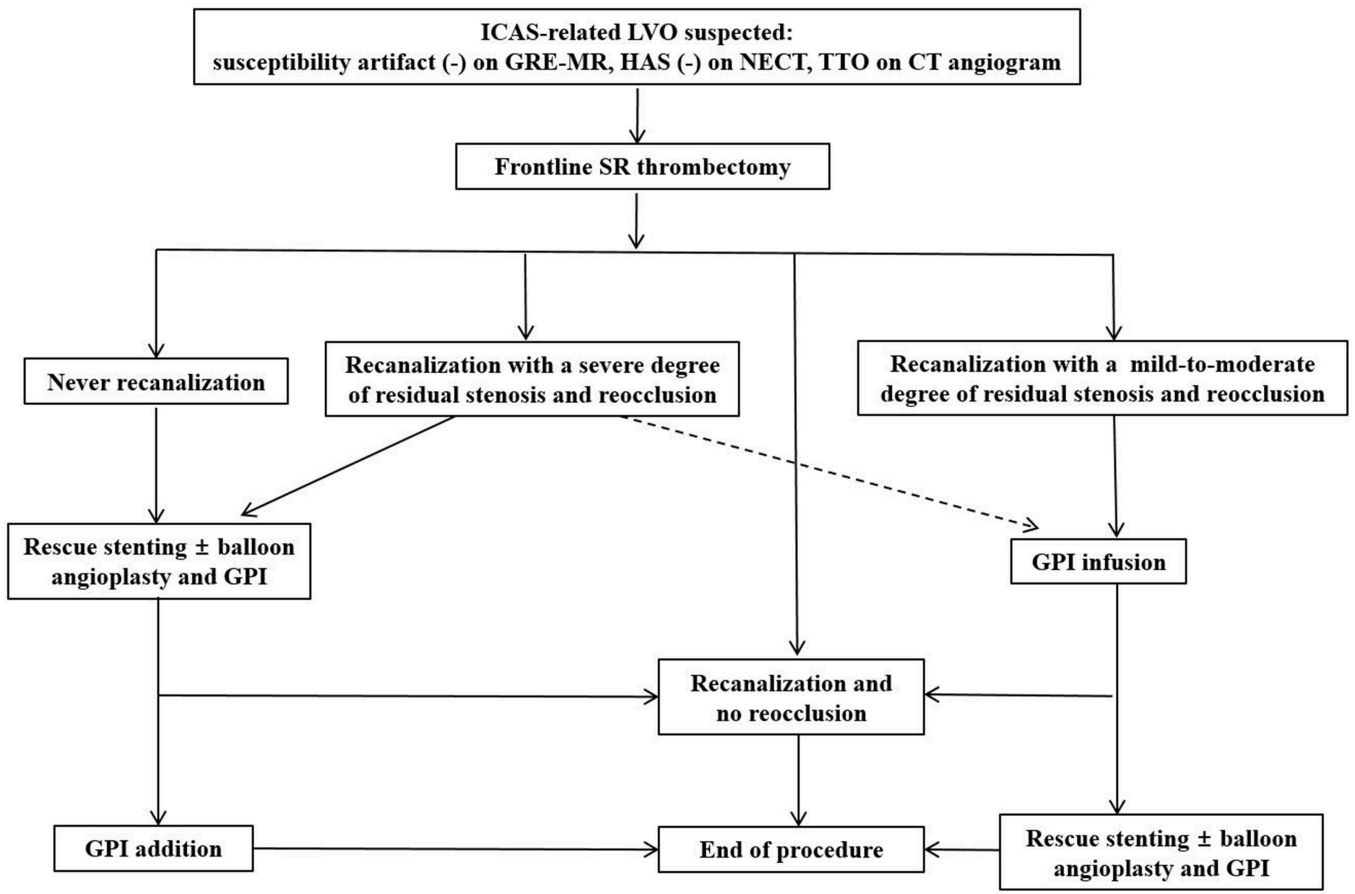
The stepwise endovascular strategy appropriate for intracranial atherosclerotic stenosis-related large vessel occlusion. ICAS, intracranial atherosclerotic stenosis; LVO, large vessel occlusion; GRE-MR, gradient echo magnetic resonance imaging; HAS, hyperdense artery sign; NECT, non-enhanced computed tomography; TTO, truncal type occlusion; CT, computed tomography; SR, stent retriever; GPI, glycoprotein IIb/IIIa inhibitor.

From practical point of view, a microcatheter with inner diameter 0.021-inch may be less difficult in delivery to the target lesion of ICAS-related LVO than a large-bore aspiration catheter because the relevant parent artery is likely more tortuous in ICAS-related LVO than in embolic LVO. Furthermore, SR is better to detect ICAS-related LVO by showing occlusion type (truncal or branching-site) as well as to obtain first-pass recanalization (Table 3). Therefore, SR thrombectomy is recommended as frontline modality if ICAS-related LVO is suspected. After achieving initial recanalization, a follow-up angiogram should be obtained every 10 min at least up to 30 min for detecting reocclusion (tendency). If reocclusion occurs with mild-to-moderate degree of residual stenosis after initial recanalization, GPI should be first recommended for avoiding acute stenting so far as possible. Whereas, if recanalization is never obtained or if reocclusion appears due to a severe degree of residual stenosis, rescue stenting and/or balloon angioplasty may be considered (Figure 4).

**Table 3:** Comparison between stent retriever and contact aspiration thrombectomy in practice.

	**Stent retriever**	**Contact aspiration**
Delivery to the target lesion	Not difficult	Occasionally difficult
Help in differentiation in ICAS-related from embolic LVO during the procedure (7, 13)^*^	Yes	No
First-pass recanalization success (23)	Higher	Lower

* *, by disclosing occlusion type (truncal-type or branching-site occlusion). Truncal-type is suggestive of intracranial atherosclerosis related large vessel occlusion*.

Although there has been no well-controlled study yet, rescue stenting and/or GPI use may be recommended as a rescue endovascular strategy appropriate for ICAS-LVO with never recanalization or repeat reocclusion. A prospective study is needed to find most appropriate endovascular strategy for ICAS-related LVO.

## Conclusions

Recanalization success and puncture-to-recanalization (procedure) time are two important procedural prognostic factors in ICAS-related LVO. With similar recanalization rate and procedure time, clinical outcome of ICAS-related LVO would be comparable to that of embolic LVO. For obtaining successful recanalization in ICAS-related LVO as fast and with high rate as in embolic LVO, the specific EVT strategy appropriate for ICAS is needed.

## Author Contributions

BK established the study idea, designed the manuscript structure, acquired and analyzed the data, and wrote the manuscript. J-HB and HP established the study idea, designed the manuscript structure, and made critical revisions to the manuscript with substantive intellectual content.

### Conflict of Interest Statement

The authors declare that the research was conducted in the absence of any commercial or financial relationships that could be construed as a potential conflict of interest.

## References

[1] Powers WJ Rabinstein AA Ackerson T Adeoye OM Bambakidis NC Becker K. Guidelines for the early management of patients with acute ischemic stroke: a guideline for healthcare professionals from the American Heart Association/American Stroke Association. Stroke. (2018) 49:e46–110. 10.1161/STR.0000000000000158 29367334

[2] Goyal M Menon BK van Zwam WH Dippel DW Mitchell PJ Demchuk AM Endovascular thrombectomy after large-vessel ischemic stroke: a meta-analysis of individual patient data from five randomized trials. Lancet. (2016) 387:1723–31. 10.1016/S0140-6736(16)00163-X 26898852

[3] Lapergue B Blanc R Gory B Labreuche J Duhamel A Marnat G. Effect of endovascular contact aspiration vs stent retriever on revascularization in patients with acute ischemic stroke and large vessel occlusion: the ASTER randomized clinical trial. JAMA. (2017) 318:443–52. 10.1001/jama.2017.9644 28763550 PMC5817613

[4] Nogueira RG Jadhav AP Haussen DC Bonafe A Budzik RF Bhuva P. Thrombectomy 6 to 24 hours after stroke with a mismatch between deficit and infarct. N Engl J Med. (2018) 378:11–21. 10.1056/NEJMoa1706442 29129157

[5] Albers GW Marks MP Kemp S Christensen S Tsai JP Ortega-Gutierrez S Thrombectomy for stroke at 6 to 16 hours with selection by perfusion imaging. N Engl J Med. (2018) 22:708–18. 10.1056/NEJMoa1713973 PMC6590673 29364767

[6] Kim BM. Causes and solutions of endovascular treatment failure. J Stroke. (2017) 19:131–42. 10.5853/jos.2017.00283 28592777 PMC5466284

[7] Baek JH Kim BM Kim DJ Heo JH Nam HS Song D. Importance of truncal-type occlusion in stentriever-based thrombectomy for acute stroke. Neurology. (2016) 87:1542–50. 10.1212/WNL.0000000000003202 27629085

[8] Kang DH Kim YW Hwang YH Park SP Kim YS Baik SK. Instant reocclusion following mechanical thrombectomy of in situ thromboocclusion and the role of low-dose intra-arterial tirofiban. Cerebrovasc Dis. (2014) 37:350–5. 10.1159/000362435 24941966

[9] Wong LK. Global burden of intracranial atherosclerosis. Int J Stroke. (2006) 1:158–9. 10.1111/j.1747-4949.2006.00045.x 18706036

[10] Bang OY. Intracranial atherosclerosis: current understanding and perspectives. J Stroke. (2014) 16:27–35. 10.5853/jos.2014.16.1.27 24741562 PMC3961814

[11] Toyoda K Koga M Hayakawa M Yamagami H Acute reperfusion therapy and stroke care in Asia after successful endovascular trials. Stroke. (2015) 46:1474–81. 10.1161/STROKEAHA.115.008781 25944322

[12] Lee JS Hong JM Lee KS Suh HI Choi JW Kim SY. Primary stent retrieval for acute intracranial large artery occlusion due to atherosclerotic disease. J Stroke. (2016) 18:96–101. 10.5853/jos.2015.01347 26467196 PMC4747073

[13] Baek JH Kim BM Yoo J Nam HS Kim YD Kim DJ. Predictive value of computed tomography angiography-determined occlusion type in stent retriever thrombectomy. Stroke. (2017) 48:2746–52. 10.1161/STROKEAHA.117.018096 28864601

[14] Baek J-H Kim BM Heo JH Kim DJ Nam HS Kim YD. Outcomes of endovascular treatment for acute intracranial atherosclerosis-related large vessel occlusion. Stroke. (2018) 49:2699–705. 10.1161/STROKEAHA.118.022327 30355204

[15] Yoon W Kim SK Park MS Kim BC Kang HK. Endovascular treatment and the outcomes of atherosclerotic intracranial stenosis in patients with hyperacute stroke. Neurosurgery. (2015) 76:680–6. 10.1227/NEU.0000000000000694 25988927

[16] Lee JS Hong JM Lee KS Suh HI Demchuk AM Hwang YH. Endovascular therapy of cerebral arterial occlusions: intracranial atherosclerosis versus embolism. J Stroke Cerebrovasc Dis. (2015) 24:2074–80. 10.1016/j.jstrokecerebrovasdis.2015.05.003 26163890

[17] Al Kasab S Almadidy Z Spiotta AM Turk AS Chaudry MI Hungerford JP. Endovascular treatment for AIS with underlying ICAD. J Neurointerv Surg. (2017) 9:948–51. 10.1136/neurintsurg-2016-012529 27502403

[18] Jia B Feng L Liebeskind DS Huo X Gao F Ma N. Mechanical thrombectomy and rescue therapy for intracranial large artery occlusion with underlying atherosclerosis. J Neurointerv Surg. (2018) 10:746–50. 10.1136/neurintsurg-2017-013489 29203731

[19] Lee JS Lee S-J Yoo JS Hong J-H Kim C-H Kim Y-W. Prognosis of acute intracranial atherosclerosis-related occlusion after endovascular treatment. J Stroke. (2018) 20:394–403. 10.5853/jos.2018.01627 30309234 PMC6186924

[20] Matias-Guiu JA Serna-Candel C Matias-Guiu J. Stroke etiology determines effectiveness of retrievable stents. J Neurointerv Surg. (2014) 6:e11. 10.1136/neurintsurg-2012-010395 22591732

[21] Gascou G Lobotesis K Machi P Maldonado I Vendrell JF Riquelme C. Stent retrievers in acute ischemic stroke: complications and failures during the perioperative period. AJNR Am J Neuroradiol. (2014) 35:734–40. 10.3174/ajnr.A3746 24157734 PMC7965801

[22] Libby P. Inflammation in atherosclerosis. Nature. (2002) 420:868–74. 10.1038/nature01323 12490960

[23] Kang DH Yoon W Kim SK Baek BH Lee YY Kim YW Endovascular treatment for emergent large vessel occlusion due to severe intracranial atherosclerotic stenosis. J Neurosurg. (2018) 1:1–8. 10.3171/2018.1.JNS172350 29932374

[24] Baek JH Kim BM Kim DJ Heo JH Nam HS Yoo J. Stenting as a rescue treatment after failure of mechanical thrombectomy for anterior circulation large artery occlusion. Stroke. (2016) 47:2360–3. 10.1161/STROKEAHA.116.014073 27444259

[25] Chang Y Kim BM Bang OY Baek JH Heo JH Nam HS. Rescue stenting for failed mechanical thrombectomy in acute ischemic stroke: a multicenter experience. Stroke. (2018) 49:958–64. 10.1161/STROKEAHA.117.020072 29581342

[26] Hwang Y-H Kim Y-W Kang D-H Kim Y-S Liebeskind DS Impact of target residual stenosis on outcome after endovascular revascularization. Stroke. (2016) 47:1850–7. 10.1161/STROKEAHA.116.013046 27174525 PMC4927379

[27] Papanagiotou P Haussen DC Turjman F Labreuche J Piotin M Kastrup A. Carotid stenting with antithrombotic agents and intracranial thrombectomy leads to the highest recanalization rate in patients with acute stroke with tandem lesions. JACC Cardiovas Intervent. (2018) 11:1290–9. 10.1016/j.jcin.2018.05.036 29976365

[28] Kim SK Yoon W Kim TS Heo TW Park MS. Histologic analysis of retrieved clots in acute ischemic stroke: correlation with stroke etiology and gradient-echo MRI. AJNR Am J Neuroradiol. (2015) 36:1756–62. 10.3174/ajnr.A4402 26159515 PMC7968760

[29] Kim SK Baek BH Lee YY Yoon W. Clinical implications of CT hyperdense artery sign in patients with acute middle cerebral artery occlusion in the era of modern mechanical thrombectomy. J Neurol. (2017) 264:2350–456. 10.1007/s00415-017-8655-0 29075836

